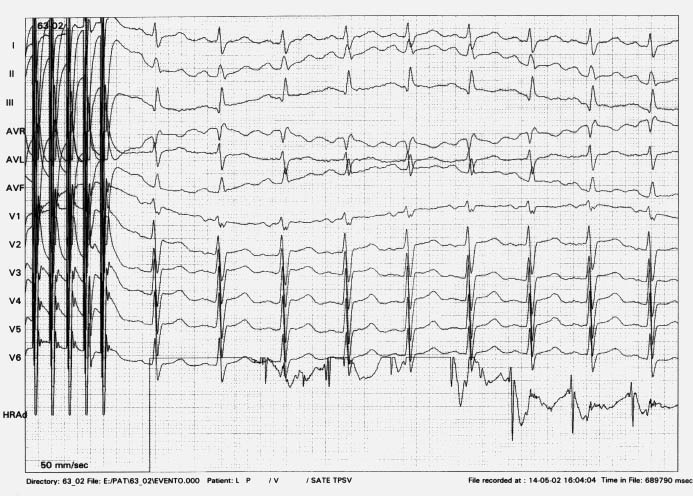# Intra-atrial Re-entrant Tachycardia with Wenckebach Periodicity

**Published:** 2003-10-01

**Authors:** Berardo Sarubbi, Pasquale Vergara, Michele D'Alto, Francesco Sessa, Raffaele Calabro

**Affiliations:** Second University of Naples, Chair of Cardiology, GUCH Unit, Division of Paediatric Cardiology, Monaldi Hospital

A 15-year-old girl, previously asymptomatic for palpitations, underwent a successful atrial septal defect (ASD) device closure. Twelve weeks after the procedure, the patient was admitted complaining of dyspnoea on effort and palpitations. The twelve-lead ECG showed a narrow QRS tachycardia with slight heart rate irregularity, with a mean HR of 170bpm. P waves were not clearly identified with a suspicious of negative P-waves in II, III and aVF leads. No clear relationship could be observed between the suspected P waves and QRS complexes (Figure 1).

Echocardiographic evaluation showed normal position of ASD Device, with no residual shunts, mild dilatation of right atrium and right ventricle with a moderate biventricular systolic function impairment. Transesophageal electrophysiological study showed an atrial tachycardia with a regular A-A interval of 220ms and an A-V conduction delay with a A-V nodal Wenckebach periodicity, leading to an irregular V-V interval ranging between 375 and 300ms (Figure 2). Overdrive atrial pacing at a cycle length of 140ms (almost 70% of the measured A-A cycle) could stop the tachycardia (Figure 3).

The case represents a rare form of intra-atrial re-entry tachycardia with an A-V nodal Wenckebach periodicity. It has been already shown that in intra-atrial re-entry tachycardia, variables degrees of block may be present throughout the entire episode of sustained tachycardia. Neither the ventricles nor the A-V node are required for this arrhythmia. It is the appearance of AV block with maintenance of the supraventricular tachycardia that strongly suggests a supranodal origin. The presence of persistent termination of the arrhythmia through atrial pacing excluded an automatic origin.

## Figures and Tables

**Figure 1:**
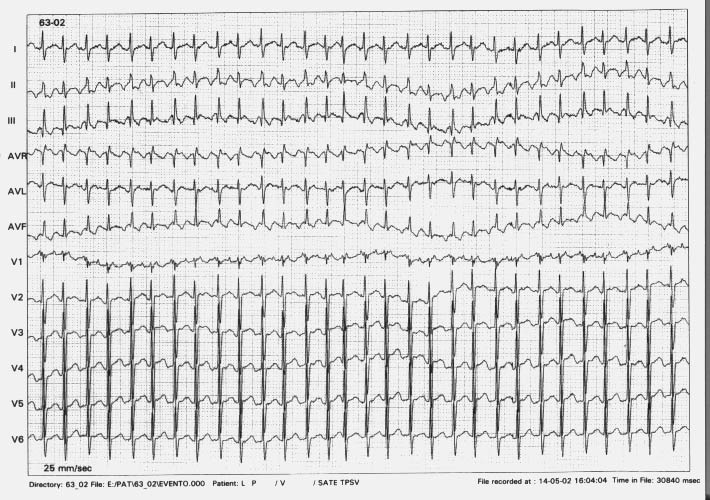


**Figure 1:**
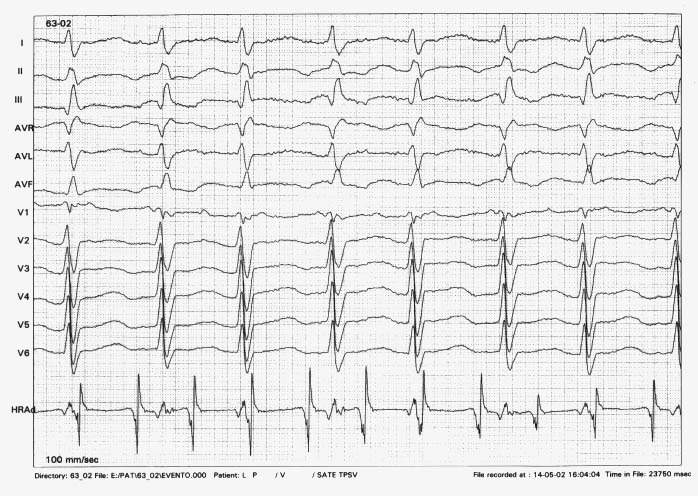


**Figure 3:**